# Bringing trees back into the human evolutionary story: recent evidence from extant great apes

**DOI:** 10.1080/19420889.2023.2193001

**Published:** 2023-03-21

**Authors:** Rhianna C. Drummond-Clarke

**Affiliations:** a Department of Human Origins, Max Planck Institute for Evolutionary Anthropology, Leipzig, Germany; b Institut für Zoologie und Evolutionsforschung, Friedrich Schiller Universität Jena, Jena, Germany

**Keywords:** Adaptation, arboreality, bipedalism, chimpanzee, great apes, hominin, human origins and evolution, savannah

## Abstract

Hypotheses have historically linked the emergence and evolution of defining human characteristics such as bipedal walking to ground-dwelling, envisioning our earliest ancestors as living in treeless savannahs (i.e. the traditional savannah hypothesis). However, over the last two decades, evidence from the fossil record combined with comparative studies of extant apes have challenged this hypothesis, instead favoring the importance of arboreality during key phases of hominin evolutionary history. Here we review some of these studies, including a recent study of savannah chimpanzees that provides the first model of how bipedalism could have been adaptive as an arboreal locomotor behavior in early hominins, even after the forests receded during the early Miocene-Pliocene transition. We suggest that whilst a shift to exploiting open habitats catalyzed hominin divergence from great apes, adaptations to arboreal living have been key in shaping what defines humans today, in counter to the traditional savannah hypothesis. Future comparative studies within and between great ape species will be instrumental to understanding variation in arboreality in extant apes, and thus the processes shaping human evolution over the last 3–7 million years.

Humans are well-adapted to bipedalism (walking on two legs). Our ape cousins, conversely, all exhibit features well-adapted for tree-climbing, such as curved fingers and long forelimbs [1]]. Hypotheses have thus historically linked human origins to a ground-dwelling environment, envisioning our earliest ancestors as living in treeless savannahs (i.e. the traditional savannah hypothesis), reviewed in [2]. However, over the last two decades, evidence from the fossil record, combined with comparative studies of extant apes (including humans) have challenged this hypothesis, instead favoring the importance of arboreality during key phases of hominin evolutionary history (e.g. [2–4]). Here, we review some recent studies that suggest that whilst a shift to exploiting open habitats catalyzed hominin divergence from great apes, adaptations to arboreal living have been key in shaping what defines humans today.

## Fossil evidence

The presence of skeletal morphology associated with bipedalism has been a defining feature separating the earliest hominins from other fossil apes [5]. However, a porous fossil record leaves much uncertainty surrounding the context and timing of the emergence and evolution of bipedalism. Complicating our reconstructions of this transition, early hominin species with morphological adaptations to bipedalism retain morphology advantageous for arboreal locomotion, such as curved fingers and long forelimbs [e.g., *Ardipithecus* [6,7], *A. afarensis* [8]]. Some argue that these arboreal adaptations are ‘useless remnants’ from an arboreal past, assuming that morphological adaptations to bipedal walking, such as rigid ankles and feet, are disadvantageous for arboreal locomotor behavior (e.g [9; 10]). However, isotopic [11–13] and dental [12,14] evidence from early hominins highlights a chimpanzee-like diet, high in arboreal food sources. Furthermore, several late-surviving hominins also had forelimbs with morphology associated with arboreal locomotion [e.g., *Homo naledi* [15], *Homo floresiensis* [16]] suggests that climbing in an arboreal environment was a significant component of the daily lives of at least some species throughout much of hominin evolution.

Moreover, palaeohabitat reconstructions of fossil hominin sites repeatedly place early hominins in savannah-mosaic habitats [e.g., *Orrorin* [17], *Ardipithecus* [7,18], and *Au. afarensis* [19]], rather than the treeless grasslands suggested by the historical savannah hypothesis. Savannah-mosaics, although indeed more arid and open than rainforests, are still substantially wooded and include forest strips [20–22], lending support to the notion (and importantly the possibility) that bipedalism may have been useful for tree living, as well as on the ground.

## Extant ape studies

Whereas fossil and paleoenvironmental evidence suggests that early hominins lived in wooded savannah habitats, studies of extant primates provide a contemporary perspective of the behaviors possible in such contexts (see also [21,23]). Quantitative, observational studies of locomotor ecology of wild, extant primates (e.g [24; 25]) and, in particular, great apes (e.g [20; 26; 27] provide valuable insights into how and why bipedalism may have evolved in a large-bodied fossil ape. For example, wild orangutans typically used bipedalism to navigate flexible terminal branches in the canopy, suggesting bipedal locomotion may have evolved initially in the trees [3,27]. Alternatively, wild chimpanzees living in forest habitats typically use a bipedal posture either on the ground [26,28] or branches [29] to reach foods within the trees, suggesting that bipedalism evolved first as a feeding posture, before being used as a locomotor behavior on the ground.

Chimpanzees not only live in forests, but across an ecological gradient that includes savannah woodlands. In light of hominin palaeohabitat reconstructions, combined with chimpanzees being our closest living relative and sharing a similar brain size and forelimb morphology with early hominins, the importance of these open-habitat chimpanzee communities for modeling early hominin behavior has been recognized since the early 1990s (e.g [30; 31]). In a recent study on the positional behavior of such savannah-dwelling chimpanzees from the Issa valley, authors found that compared to forest dwelling communities, Issa chimpanzees were highly arboreal during locomotion, despite living in a more open habitat [20]. In addition, bipedalism remained an arboreal behavior and was used more for locomotion compared with that of forest-dwelling chimpanzees [20]. These findings provide the first model of how bipedalism could have been adaptive as an arboreal locomotor behavior in early hominins, even after the forests receded during the early Miocene-Pliocene transition.

There is also emerging evidence that arboreal living may have promoted a key element of the human vocal repertoire: consonant-like sounds. Lameira [32] has argued that human speech may have “come from above”, with ground-living later allowing richer gestural, hand-to-tool, or postural repertoires. Combined, the proposed arboreal origins of bipedalism and consonant-like sounds demand a reconsideration of the importance of arboreal living in human evolution.

Finally, data from modern humans further support the idea that hominins did not have to sacrifice their climbing ability to become efficient terrestrial bipeds. For example, human hunter-gatherers who climb trees regularly achieve extraordinary ankle dorsiflexion equivalent to that seen in chimpanzees during climbing through soft tissue adaptations [33]. Further, an experimental study of modern humans in a simulated, unstable arboreal environment showed ‘light touch’ support by the fingertips significantly enhanced balance and reduced muscle activity, suggesting the short fingers of the human hand may have been first advantageous for arboreal bipedal locomotion before enhancing manipulation and tool use [34].

## Why stay in the trees?

Morphological and behavioral evidence notwithstanding, there remains the question of why arboreal living in hominins may have persisted in the face of receding forests and expanding savannah woodlands. Savannah habitats are associated with more sparsely distributed food sources [21], which may have selected for increased terrestrial travel in our ancestors (e.g [31; 35]). Our observations of Issa chimpanzees suggest an alternative. During the dry season at Issa, for example, chimpanzees feed on *Brachystegia* sp [36,37] – a woodland species that dominates the landscape and when in fruit, provides an abundant, woodland food source in adjacent trees with wide crowns. Could the high concentration of preferred feeding trees in fact facilitate an arboreal lifestyle despite overall fewer trees? Another hypothesis is that during times of food scarcity, Issa chimpanzees may spend more time in a single tree, thus minimizing larger travel distance between feeding trees [20].

Alternatively, rather than being attracted to the trees, chimpanzees may be actively avoiding the ground. Issa, like many early hominin sites, is characterized by a diverse carnivore guild. Combined with a complex and mountainous terrain [20,38], individuals could exploit arboreal pathways to avoid terrestrial dangers (e.g. [39]). These and other drivers of arboreal savannah living demand investigation.

In summary, there are likely multiple selective pressures that promoted the evolution of bipedalism in the human lineage (e.g. [40]). Here, we suggest that these centered around selection to maintain an arboreal lifestyle in a more open habitat, and possibly to efficiently forage whilst avoiding large carnivores. Future comparative studies within and between great ape species will be instrumental to understanding variation in arboreality in extant apes, and thus the processes shaping human evolution over the last 3–7 million years.
